# Identification of Coagulation Phenotypes Using Viscoelastic and Conventional Assays During Veno‐Venous Extracorporeal Membrane Oxygenation: A Hierarchical Clustering Analysis

**DOI:** 10.1111/aor.70153

**Published:** 2026-04-21

**Authors:** Daisuke Irimada, Yudai Iwasaki, Yusuke Takei, Moe Oyama, Ami Tanaka, Shinichi Yamaguchi, Naoya Kobayashi, Daisuke Konno, Takuya Shiga, Masanori Yamauchi

**Affiliations:** ^1^ Department of Anesthesiology and Perioperative Medicine Tohoku University Graduate School of Medicine Sendai Japan; ^2^ Experience Design and Alliance Section Tohoku University Hospital Sendai Japan

**Keywords:** activated partial thromboplastin time, anticoagulation, cluster analysis, coagulopathy, extracorporeal membrane oxygenation, thromboelastography

## Abstract

**Background:**

Veno‐venous extracorporeal membrane oxygenation (V‐V ECMO) is essential for managing severe respiratory failure; however, hemorrhagic and thrombotic complications remain major challenges. Conventional activated partial thromboplastin time (APTT) monitoring may inadequately reflect the complex coagulation changes during ECMO. Although viscoelastic hemostatic assays (VHAs) provide a comprehensive assessment, discrepancy patterns between VHAs and conventional tests in V‐V ECMO patients remain unclear.

**Objectives:**

This study aimed to identify distinct coagulation phenotypes in patients receiving V‐V ECMO using hierarchical clustering of VHA and conventional coagulation parameters.

**Methods:**

This retrospective observational study was conducted at a tertiary university hospital in Japan. Patients receiving V‐V ECMO between November 2020 and June 2023 who underwent VHA and conventional coagulation testing were included. Coagulation profiles were clustered using hierarchical analysis of five variables. Cluster validity was assessed, and results were visualized using principal component analysis and generalized additive models.

**Results:**

A total of 19 patients and 110 VHA measurements were analyzed. Three distinct coagulation phenotypes were identified. Cluster 1 demonstrated concordant prolongation of APTT and citrated kaolin reaction time (CK‐R). Clusters 2 and 3 exhibited disproportionately prolonged CK‐R relative to APTT, with Cluster 3 further characterized by elevated fibrinogen and platelet counts. Anticoagulant use, laboratory markers, and clinical outcomes, including thrombotic and bleeding events, were evaluated. Complication rates did not differ significantly among clusters.

**Conclusions:**

Hierarchical clustering identified three distinct coagulation profiles among V‐V ECMO patients, highlighting the heterogeneity of ECMO‐related coagulopathy. Thromboelastography‐derived CK‐R may complement APTT monitoring to inform more tailored anticoagulation strategies in this population.

## Graphical abstract

Hierarchical clustering of viscoelastic and conventional coagulation parameters identified three distinct phenotypes in V‐V ECMO patients, revealing substantial APTT–CK‐R discordance and highlighting the value of TEG‐derived markers for detecting coagulopathy not captured by standard monitoring.

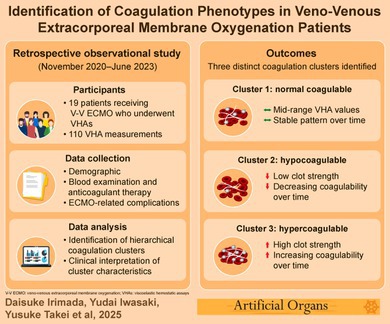

## 1 Introduction

Veno‐venous extracorporeal membrane oxygenation (V‐V ECMO) is a widely used therapeutic modality for patients with respiratory failure, including acute respiratory distress syndrome and chronic obstructive pulmonary disease, and serves as a bridge to lung transplantation in patients with chronic lung diseases [1]. During V‐V ECMO management, hemorrhagic and thrombotic complications are observed in numerous studies and are associated with increased mortality, circuit exchange frequency, and healthcare costs [2, 3]. Effective coagulation monitoring and control are central components of ECMO management. The Extracorporeal Life Support Organization guidelines recommend routine monitoring of activated partial thromboplastin time (APTT) every 6–12 h [4]. Nonetheless, APTT measurements may not always accurately reflect the coagulation profiles under the complex pathophysiology of ECMO. Several studies have reported discrepancies between APTT and anti‐Xa assays in patients receiving ECMO under systemic heparin anticoagulation. In one retrospective neonatal ECMO cohort, APTT was confounded by factor deficiency and hepatic/consumptive coagulopathy, whereas anti‐Xa was susceptible to analytical interference, yielding discordant results [5]. A prespecified secondary analysis of a prospective randomized trial in adults showed frequent APTT–anti‐Xa mismatch in association with clinical factors such as heparin resistance and higher unfractionated heparin (UFH) dosing [6]. A recent review also summarized evidence suggesting that anti‐Xa‐guided heparin management may reduce bleeding and mortality compared with APTT‐based strategies [7]. These findings suggest that the sole use of APTT during ECMO management might be associated with an increased risk of thrombotic and bleeding complications. Although the anti‐Xa assay is considered the gold standard for monitoring unfractionated heparin (UFH) [8, 9], it is not frequently used in ECMO management because of the limited accessibility, which is particularly problematic in Japan [10].

To overcome these limitations, viscoelastic hemostatic assays (VHAs), such as thromboelastography (TEG), offer whole‐blood‐based assessments that provide a more comprehensive view of coagulation profiles [11]. VHAs can capture subtle coagulation disturbances, including prolonged reaction times, which may not be detected by standard coagulation assays, such as APTT. Notably, VHA tracings showing “flat‐lines,” indicating unmeasured prolonged citrated kaolin reaction time (CK‐R), have been documented in patients receiving ECMO despite normal or mildly elevated APTT values [12], suggesting potential discrepancies between conventional and viscoelastic markers. When APTT–anti‐Xa discordance or unexplained bleeding occurs, VHA may provide complementary information concerning clotting time and strength. Although VHA enables meticulous coagulation monitoring, it is a costlier analytical approach than traditional blood tests [13]. Thus, its application needs to be optimized by identifying the clinical or laboratory patterns that predict VHA abnormalities. To better understand these abnormalities, unsupervised clustering techniques may offer a data‐driven method for identifying latent subgroups of patients with coagulopathy, as clarified by VHA measurements. By applying hierarchical clustering to coagulation‐related variables, including CKR and APTT, patients can be classified into phenotypes that reflect distinct coagulation abnormalities.

This study aimed to identify distinct coagulation phenotypes using hierarchical cluster analysis in patients receiving V‐V ECMO, with a particular focus on the dissociation between APTT and CK‐R.

## 2 Methods

### 2.1 Study Design and Setting

This retrospective observational study was conducted at a university hospital in Japan from November 1, 2020, to June 30, 2023. This study was approved by the Institutional Review Board and Ethics Committee of the hospital (ID: 2023‐1‐504; approved on September 27, 2023) and was conducted in accordance with the principles of the Declaration of Helsinki. The requirement for written informed consent was waived because of the retrospective study design.

### 2.2 Patient Inclusion and Exclusion Criteria

Patients requiring V‐V ECMO support who underwent VHAs at least once during the support were eligible for inclusion. VHAs were obtained at the physician's discretion without prespecified criteria. Although the indication for each examination was not systematically determined in our ICU, VHAs are typically used in routine clinical practice for two purposes: (1) baseline assessment early after ECMO initiation and (2) intermittent checks prompted by bleeding concern, despite normal coagulation parameters. Patients receiving veno‐arterial ECMO, intra‐aortic balloon pumping, or left ventricular assist devices (including peripheral or external types) were excluded because of the frequent use of antiplatelet agents and differing anticoagulation profiles compared with V‐V ECMO [14]. Patients without any form of extracorporeal life support or those receiving extracorporeal carbon dioxide removal were also excluded. All ICU VHA tests during the study period were queried and then intersected with the ECMO registry to identify V‐V ECMO patients who underwent VHA.

### 2.3 Data Collection

#### 2.3.1 Demographic Information

Patient demographic data included age, height, weight, body mass index, admission type (emergent surgery, scheduled surgery, or emergent medical admission), Acute Physiology and Chronic Health Evaluation II score [15], Sequential Organ Failure Assessment score [16], underlying cause of respiratory failure leading to V‐V ECMO, chronic organ insufficiency, and additional intensive care unit (ICU) therapy during ICU admission obtained from the Japanese Intensive care PAtient Database [17]. Duration of ECMO, ICU stay, and clinical outcomes were also collected. All such data were obtained on the day of ICU admission.

#### 2.3.2 Blood Examination Data and Anticoagulant Therapy

All VHA and conventional blood examination data were collected from each patient on the day of the VHA measurement. The timing of the VHA measurement was conducted at the physician's discretion, such as routine monitoring and the detection of coagulopathy causes. VHA was measured using thromboelastography 6s (TEG6s) (Haemonetics Corporation, USA), a cartridge‐based viscoelastic hemostasis analyzer that allows fully automated assessment of whole blood coagulation using microfluidic resonance technology [18]. The TEG6s Global Hemostasis cartridge simultaneously performs four parallel assays: citrated kaolin (CK), citrated kaolin with heparinase (CKH), citrated rapid thromboelastography (CRT), and citrated functional fibrinogen (CFF), each targeting different aspects of the coagulation cascade. In this study, the CK assay was used to determine five core parameters: R time, K time, alpha angle, maximum amplitude (MA), and LY30 (%). The CKH assay was used to assess the presence of the heparin effect, CRT for rapid global assessment of coagulation, and CFF to evaluate the contribution of fibrinogen to clot strength. “Prolonged reaction time” was defined as CK‐R of 24–59 min, based on prior literature suggesting that CK‐R values exceeding approximately 24 min during ECMO management may indicate clinically relevant coagulopathy [19], while “flat‐line” was defined as CK‐R > 60 min, according to previous literature [20]. Conventional blood examination data included platelet counts, fibrinogen, antithrombin III, APTT, and prothrombin time‐international normalized ratio. APTT was categorized into four groups: < 40, 40–60, 60–80, and > 80 s, based on the therapeutic range recommended in previous reports [4, 21]. The type of anticoagulant medication and, for heparin, the weight‐normalized dose (units/kg/h) during the 24 h before and after each VHA measurement were recorded. Blood examination and anticoagulant therapy data were collected for each patient in accordance with the VHA measurement day. As the unit of analysis was each VHA measurement rather than each patient, patients with multiple VHA measurements during ECMO were included, and all corresponding laboratory and clinical data were collected for each VHA measurement. No predefined protocol was utilized for heparin dose adjustment based on the VHA results, and the doses were recorded before and after VHA to reflect the actual clinical practice during routine management. Anti‐Xa monitoring was not available at our institution and was therefore not performed during the study period.

#### 2.3.3 Extracorporeal Membrane Oxygenation‐Related Complications

According to the timing of VHA measurement, the following ECMO‐associated outcomes were collected: (1) circuit exchange due to thrombosis, membrane deterioration, coagulopathy, and plasma leak and (2) bleeding complication, classified from types 0 to 4 (type 0, no bleeding; type 1, any overt bleeding requiring heparin infusion rate reduction or packed red blood cell transfusion [provided hemoglobin drop was related to bleeding]; type 2, any overt bleeding that required both heparin infusion rate reduction and packed red blood cell transfusion [provided hemoglobin drop was related to bleeding]; type 3, any life‐threatening bleeding that required packed red blood cell transfusion and surgical intervention for control of bleeding or ECMO discontinuation; type 4, any fatal bleeding) [22]. These data were collected for exploratory analysis, with the aim of illustrating any potential temporal relationships between coagulation phenotype and complications within a limited observation window, defined by the routine 24‐h VHA assessment interval, with outcomes assessed within ±24 h of each measurement.

### 2.4 Group Allocation Using a Hierarchical Cluster Analysis

Agglomerative hierarchical cluster analysis was performed to identify the latent patterns of coagulation abnormalities. Prior to clustering, residuals were calculated from a linear regression model that predicted CK‐R time from APTT to quantify the degree of disproportionate CK‐R prolongation. This residual represents the excess clotting time beyond that expected from the APTT value and can be interpreted as an indicator of heparin‐independent or factor‐related coagulopathy not captured by conventional testing. In addition to these residuals, the CK‐R/APTT ratio was calculated as a secondary indicator to highlight relative CK‐R prolongation beyond that expected from APTT alone. These two derived metrics were combined with three conventional coagulation parameters (APTT, fibrinogen level, and platelet count) to form a five‐dimensional dataset. Multicollinearity among the variables was assessed before clustering using variance inflation factors (VIFs), all of which were below 10, indicating no problematic collinearity [23]. Each variable was standardized, and the pairwise Euclidean distances between the observations were computed. Ward's linkage method was then applied to iteratively merge similar patients into clusters, minimizing within‐cluster variance at each step.

To determine the optimal number of clusters, the elbow method by plotting the total within‐cluster sum of squares against the number of clusters and selecting the point at which the marginal gain in cohesion sharply decreased [24]. Cluster validity and robustness were evaluated using complementary approaches including silhouette analysis [25], the gap statistic [26], and bootstrap resampling [27]. Silhouette coefficients were computed to assess the degree of cluster separation, the gap statistic was used to compare within‐cluster dispersion against a reference null distribution, and cluster stability was further examined by calculating Jaccard similarity indices across 500 bootstrap iterations.

Principal component analysis (PCA) was also performed using the standardized input variables to visually assess the separation of the identified clusters. The first two principal components were extracted to illustrate the distribution of clusters in two‐dimensional space, and the proportion of variance explained by each component was calculated to evaluate the adequacy of this low‐dimensional representation.

To assess the stability and within‐patient consistency of the derived clustering structure, the proportion of between‐ and within‐patient variation in the cluster membership was then quantified using intraclass correlation coefficients (ICC) [28, 29]. The overall ICC was estimated using a linear mixed‐effects model with patient identifiers as random intercepts, and cluster‐specific ICCs were obtained using logistic mixed‐effects models that treated cluster membership as a binary outcome. All models were fitted using the lme4 package in R [30]. ICC values were interpreted according to established guidelines, with values of < 0.5 considered poor, 0.5–0.75 moderate, 0.75–0.9 good, and > 0.9 excellent in terms of reliability [31]. These analyses were conducted to confirm that the clustering captured both inter‐ and intra‐patient variance components before proceeding to comparative analyses.

### 2.5 Statistical Analyses

Continuous variables are presented as means ± standard deviations or medians (interquartile ranges [IQRs]), depending on their distribution, and categorical variables are expressed as percentages. Patient characteristics were described, and the time course of VHA measurements in each patient was visualized. The frequency of prolonged CK‐R stratified by APTT category was visualized to examine whether conventional coagulation markers adequately reflect VHA abnormalities. After performing the hierarchical cluster analysis, we created the following visualizations:

1. Box plots of APTT, CK‐R, CK‐R/APTT ratio, fibrinogen level, platelet count, and CK‐R residuals to compare the distribution of coagulation‐related variables across clusters.
2. Smoothed trend lines using generalized additive models with restricted cubic splines to illustrate the relationship between APTT and CK‐R within each cluster.

VHA data, blood examination data, pharmacological information, and outcome data were compared across clusters using analysis of variance or the Kruskal–Wallis test for continuous variables and the Fisher test for categorical values. Changes in anticoagulant dosing before and after VHA measurements were also analyzed across clusters. Statistical significance was defined as a two‐sided *p*‐value < 0.05. All analyses were performed using the R software (version 4.3.1) (2023‐06‐16).

## 3 Results

**FIGURE 1:**
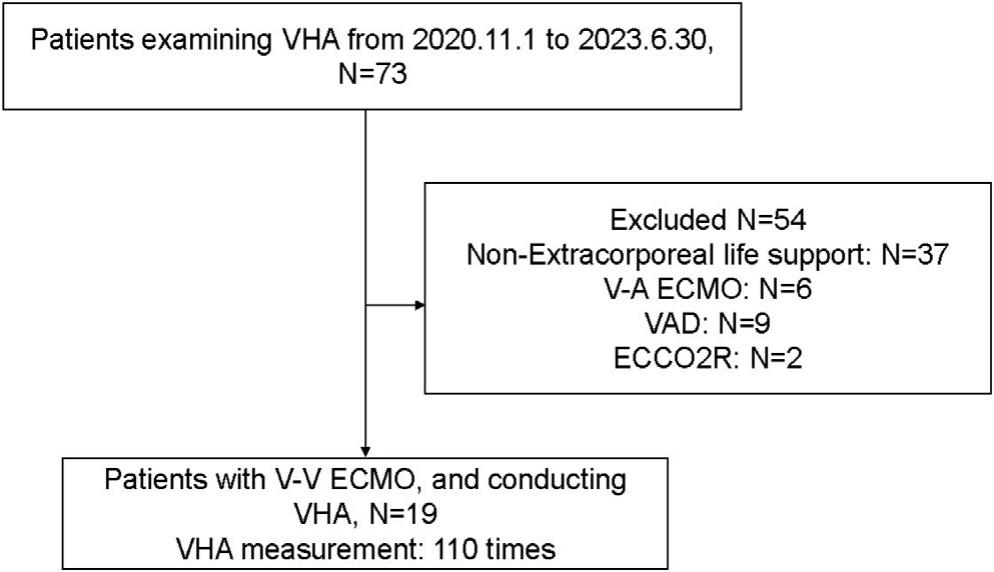
Patients' flowchart.

**FIGURE 2:**
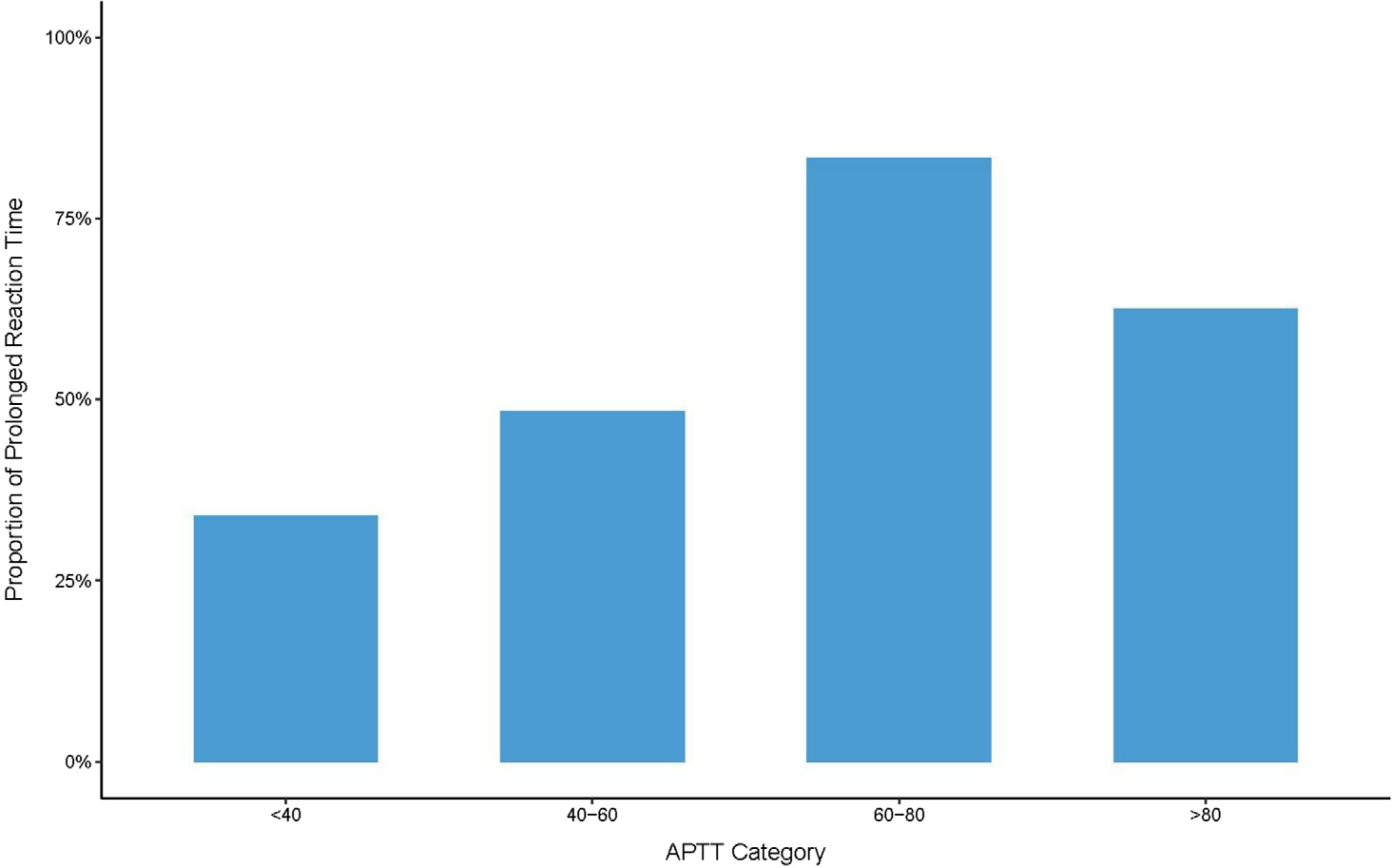
The proportion of prolonged reaction time across activated partial thromboplastin time category. [Color figure can be viewed at wileyonlinelibrary.com]

From November 1, 2020, to June 30, 2023, 73 initial patients were initially screened; 54 were excluded, yielding a final cohort of 19 patients and 110 VHA data points (Figure 1). Table 1 presents the patient characteristics. The main indication for V‐V ECMO was coronavirus disease 2019 (COVID‐19) (47.4%), and the median of V‐V ECMO was 12 days (IQR, 9–26.5) days. VHA measurement was conducted six times per patient (IQR, 2.5–7.0), and the in‐hospital mortality rate was 31.6%. Figure S1 shows the timing of the VHA measurements in each patient. Each patient exhibited a different proportion of prolonged CK‐R. Figure 2 demonstrates the proportion of patients with prolonged CK‐R according to APTT category. In all APTT categories except for ≤ 40 s, approximately 50% of the measurements demonstrated prolonged CK‐R.

**TABLE 1:** Patient characteristics.

Variables	*n* = 19
Demographic information	
Age, years (median [IQR])	56 [40.5–67.0]
Male, *n* (%)	16 (84.2)
Height, cm (median [IQR])	168.0 [161.0–174.0]
Weight, kg (median [IQR])	71.5 [60.3–89.7]
Body mass index, kg/m^2^ (median [IQR])	25.2 [23.1–31.1]
Admission type, *n* (%)	
Emergent surgery	3 (15.8)
Scheduled surgery	3 (15.8)
Emergent medical admission	13 (68.4)
Severity of patients	
APACHE II score (median [IQR])	21.00 [18.00–23.00]
SOFA score (median [IQR])	9.00 [7.50–10.50]
Underlying cause of respiratory failure leading to V‐V ECMO, *n* (%)	
COVID‐19	9 (47.4)
Hematological malignancy	3 (15.8)
Abdominal infection	2 (10.5)
Malignancy	1 (5.3)
Post‐cardiac surgery	2 (10.5)
Post‐lung transplant surgery	2 (10.5)
Complications at admission, *n* (%)	
Acute myeloid leukemia	2 (10.5)
Heart failure	1 (5.3)
Lymphoma	1 (5.3)
Cancer metastasis	1 (5.3)
Use of immunosuppressants	5 (26.3)
Additional therapy during ICU admission, *n* (%)	
Continuous renal replacement therapy	7 (36.8)
Plasma exchange	2 (10.5)
Information	
ECMO use, days (median [IQR])	12.0 [9.0–26.5]
Number of VHA measurement (median [IQR])	6.0 [2.5–7.0]
ICU stay, days (median [IQR])	37.0 [23.5–66.0]
Outcome, *n* (%)	
Death	6 (31.6)
Discharge home	7 (36.8)
Transfer to another hospital after decannulation	6 (31.6)

Abbreviations: APACHE II, Acute Physiology and Chronic Health Evaluation II; ICU, intensive care unit; IQR, interquartile range; SOFA, Sequential Organ Failure Assessment; VHA, viscoelastic hemostatic assay; V‐V ECMO, veno‐venous extracorporeal membrane oxygenation.

Figure S2A shows an elbow plot used to determine the optimal number of clusters. The optimal number of clusters was identified as three based on the inflection point of the curve, followed by gap statistical analysis in Figure S2B, with the modest local maximum of around *k* = 2–3 supporting the selection of three clusters as a parsimonious and interpretable solution. Multicollinearity for each variable was assessed using VIFs: APTT = 1.34, fibrinogen = 2.33, platelet count = 2.46, CK‐R residual = 5.27, and CK‐R/APTT ratio = 5.56. As all values were below the conventional threshold of 10, no problematic collinearity was identified. Figure S3A shows the hierarchical clustering dendrogram constructed using Ward's method, and Figure S3B illustrates the corresponding cluster classification in two‐dimensional space using principal component analysis. This analysis identified three distinct clusters based on coagulation profiles. The first and second principal components explained 47.1% and 27.7% of the total variance, respectively, capturing approximately 75% of the overall variability. The plot demonstrated a reasonable visual separation among the three clusters, supporting the robustness of the clustering structure. The three‐cluster solution demonstrated moderate separation (mean silhouette width = 0.34), and bootstrap Jaccard indices of 0.78, 0.54, and 0.48 were obtained for Clusters 1, 2, and 3, respectively, indicating moderate stability and reasonable robustness for the identified structure. The ICC analysis further evaluated within‐patient consistency in the cluster allocation, with the overall ICC of 0.43 suggesting poor global stability for the clustering structure. Cluster‐specific ICCs showed distinct patterns: Cluster 1 demonstrated poor consistency (ICC = 0.19), Cluster 2 showed minimal stability (ICC = 0.04), and Cluster 3 exhibited excellent consistency (ICC = 0.98).

**FIGURE 3:**
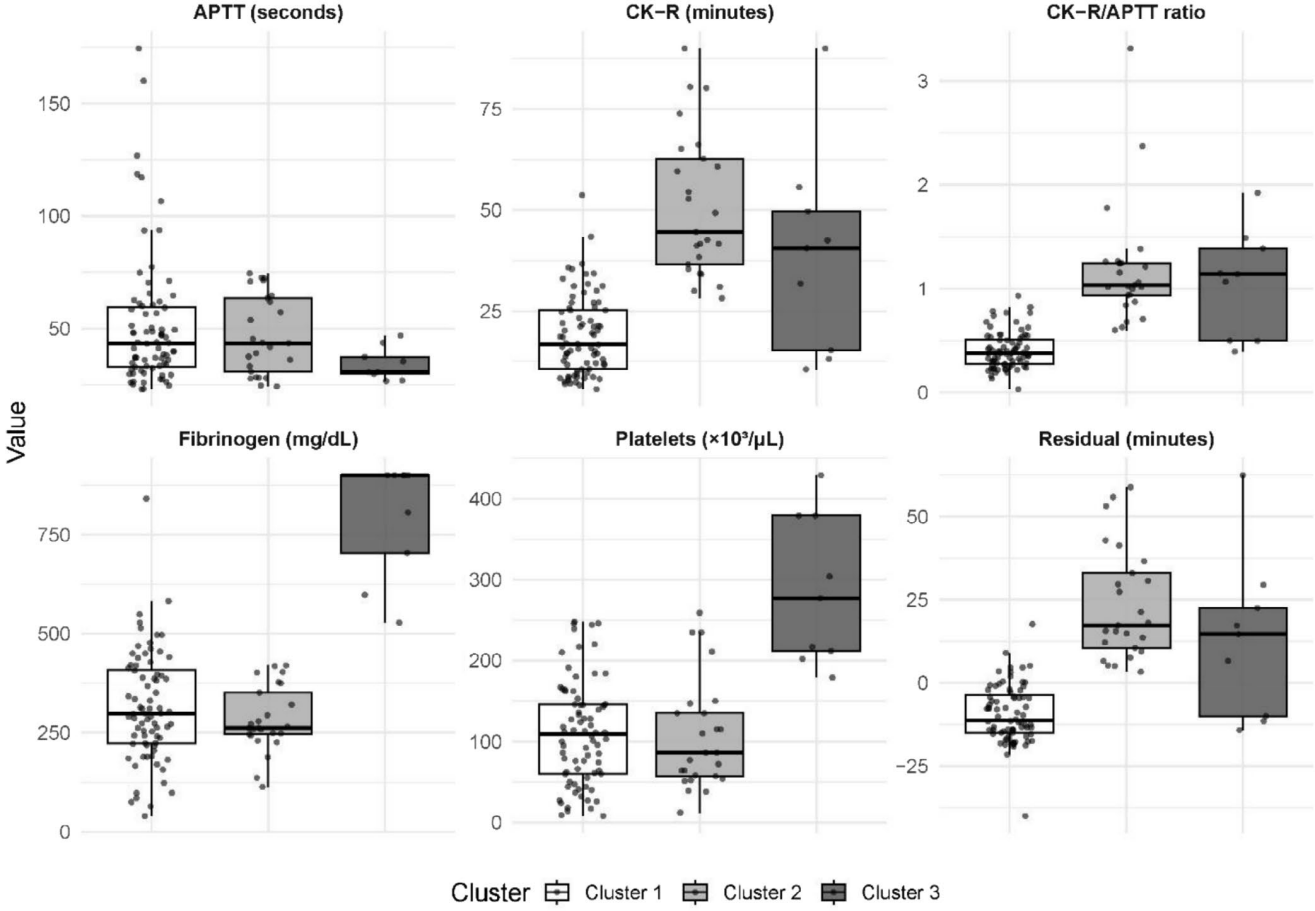
Cluster‐wise distribution of coagulation parameters.

**FIGURE 4:**
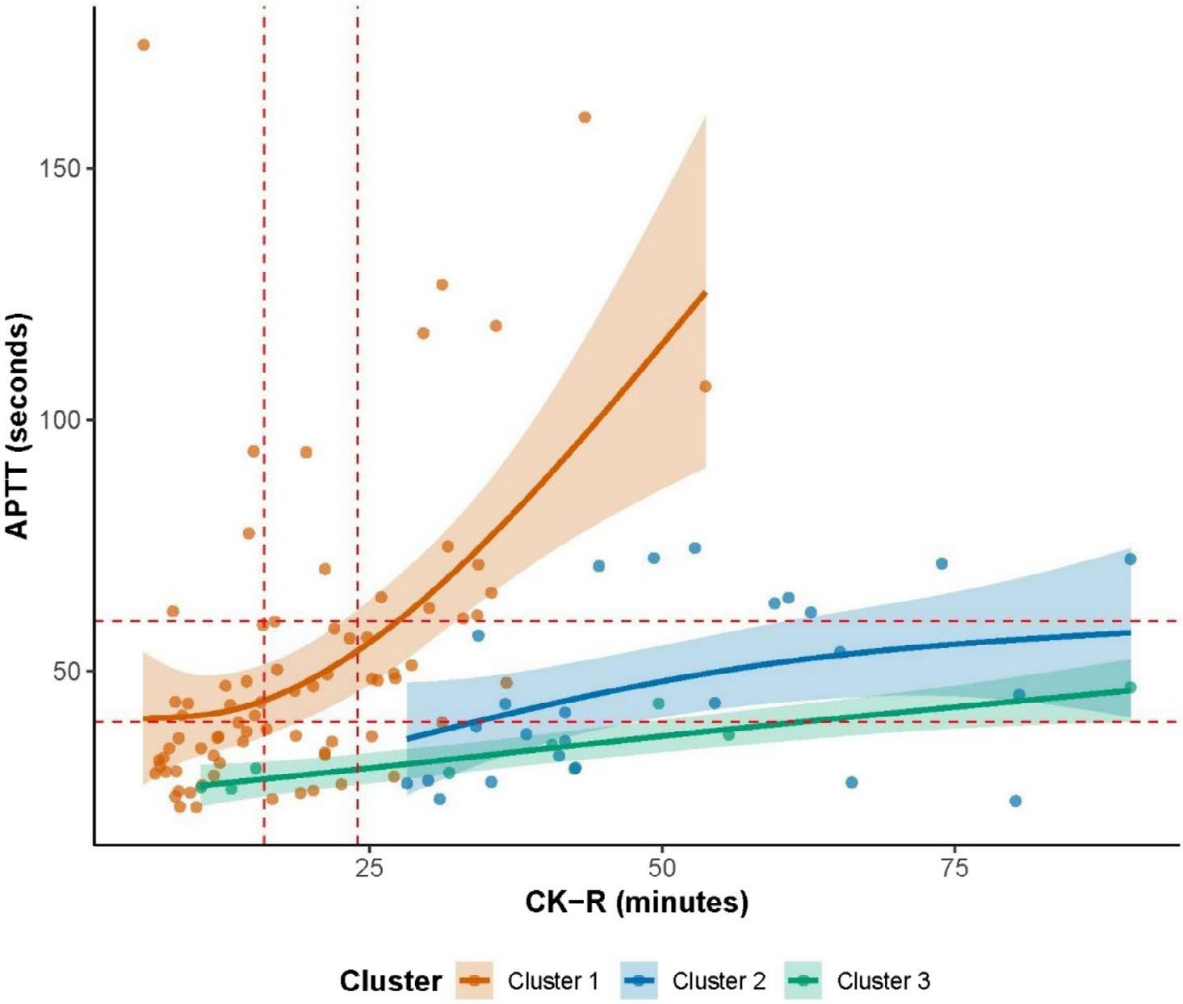
Trends in the citrated kaolin reaction and activated partial thromboplastin times, colored by cluster classification. Smoothed curves were generated using generalized additive models with restricted cubic splines to visualize the relationship between APTT and CK‐R within each cluster. [Color figure can be viewed at wileyonlinelibrary.com]

The cluster‐wise distribution of coagulation parameters is shown in Figure 3. Cluster 1 exhibited moderately prolonged APTT with corresponding CK‐R values, suggesting a relatively concordant coagulation profile. In contrast, clusters 2 and 3 demonstrated disproportionately prolonged CK‐R relative to APTT, as indicated by the elevated residuals and increased CK‐R/APTT ratios. Cluster 3 was characterized by a hypercoagulable phenotype, with markedly elevated fibrinogen levels and platelet counts. Figure 4 shows the smoothed relationship between CK‐R and APTT, stratified by cluster classification. Cluster 1 exhibited a relatively linear association between APTT and CK‐R. In contrast, clusters 2 and 3 showed disproportionate CK‐R prolongation, which was not accompanied by a corresponding increase in APTT. These smooth curves were generated using generalized additive models with restricted cubic splines and illustrate the modeled relationship between APTT and CK‐R within each cluster, providing a visual depiction of how CK‐R changed across the range of APTT values.

Table 2 presents a detailed comparison of the pharmacological information, conventional coagulation tests, and viscoelastic parameters obtained at the time of the VHA measurement across the three clusters. Clusters 1, 2, and 3 included 76 VHA data from 18 patients, 25 VHA data from 12 patients, and 9 VHA data from 3 patients, respectively (Figure S4). The primary anticoagulant was heparin; nafamostat mesylate was used in only 4/110 VHA measurements, whereas argatroban was used in 5/110 VHA measurements. Cluster 2 was particularly notable for 100% prolonged reaction time and a 32% incidence of flat‐line tracings. In addition, citrated kaolin maximum amplitude (CK‐MA) was the lowest in cluster 2. Cluster 3 exhibited the highest rate of heparin administration, along with elevated platelet counts, fibrinogen levels, and high CRT‐MA. Although statistically significant differences were observed in platelet counts and fibrinogen levels across the clusters, the median values in clusters 1 and 2 remained within the normal clinical range, suggesting limited clinical relevance, whereas cluster 3 exhibited markedly elevated levels.

**TABLE 2:** Comparison of pharmacological information, conventional coagulation tests, and viscoelastic parameters across clusters.

	Overall	Cluster 1	Cluster 2	Cluster 3	*p*
Number of VHAs	110	76	25	9	NA
Number of patients	19	18	12	3	NA
Pharmacological information at VHA measurement					
Heparin use, *n* (%)	83 (75.5)	50 (65.8)	24 (96.0)	9 (100.0)	0.002
Heparin dose before VHA measurement, units/kg/h (median [IQR])	9.4 [0.9–12.8]	5.6 [0.0–11.5]	10.2 [8.5–12.7]	19.5[15.2–21.7]	< 0.001
Heparin dose after VHA measurement, units/kg/h (median [IQR])	8.6 [1.9–12.4]	7.10 [0.0–11.7]	9.5 [6.5–11.0]	18.0 [15.2–21.7]	< 0.001
Blood examination data					
Platelet count, 10^3^/μL (median [IQR])	111.50 [60.25–163.50]	109.00 [60.00–146.00]	86.00 [57.00–135.00]	277.00 [212.00–379.00]	< 0.001
Fibrinogen, mg/dL (median [IQR])	298.00 [241.50–419.50]	298.00 [222.75–408.50]	262.00 [246.00–351.00]	900.00 [704.00–900.00]	< 0.001
AT3, % (median [IQR])	86.50 [75.50–95.00]	87.00 [71.75–95.00]	86.00 [75.00–101.00]	84.00 [79.00–92.00]	0.85
APTT, s (median [IQR])	41.50 [31.35–59.03]	43.45 [33.10–59.38]	43.50 [30.80–63.50]	30.70 [29.80–37.40]	0.109
PT‐INR (median [IQR])	1.01 [0.93–1.12]	1.00 [0.93–1.12]	1.04 [0.94–1.11]	0.95 [0.95–0.98]	0.723
d‐dimer, μg/mL (median [IQR])	39.50 [15.98–71.88]	38.95 [16.32–72.70]	45.50 [23.40–69.00]	22.20 [11.10–51.70]	0.422
APTT category (%)					0.075
< 40 s	53 (48.2)	35 (46.1)	11 (44.0)	7 (77.8)	
40–60 s	31 (28.2)	23 (30.3)	6 (24.0)	2 (22.2)	
60–80 s	18 (16.4)	10 (13.2)	8 (32.0)	0 (0.0)	
> 80 s	8 (7.3)	8 (10.5)	0 (0.0)	0 (0.0)	
VHA data					
CK					
Reaction time, min (median [IQR])	22.30 [13.12–35.70]	16.80 [10.67–25.32]	44.60 [36.60–62.70]	40.60 [15.30–49.70]	< 0.001
Maximum amplitude, mm (median [IQR])	51.40 [20.15–61.45]	55.90 [34.30–62.00]	7.10 [2.85–29.65]	62.50 [10.75–67.05]	< 0.001
LY30, % (median [IQR])	0.00 [0.00–0.00]	0.00 [0.00–0.00]	0.00 [0.00–0.00]	0.00 [0.00–0.00]	0.375
Abnormal pattern of CK‐R					
Proportion of prolonged reaction time, *n* (%)	53 (48.2)	22 (28.9)	25 (100.0)	6 (66.7)	< 0.001
Flat line, *n* (%)	9 (8.2)	0 (0.0)	8 (32.0)	1 (11.1)	< 0.001
CRT					
Reaction time, min (median [IQR])	0.50 [0.30–0.78]	0.50 [0.30–0.80]	0.50 [0.40–0.80]	0.50 [0.20–0.70]	0.603
Maximum amplitude, mm (median [IQR])	61.55 [49.73–67.02]	61.85 [49.38–66.83]	55.40 [48.20–61.80]	71.50 [69.60–75.20]	0.001
LY30, % (median [IQR])	0.00 [0.00–0.00]	0.00 [0.00–0.00]	0.00 [0.00–0.10]	0.00 [0.00–0.12]	0.096
CKH					
Reaction time, min (median [IQR])	10.45 [7.82–14.40]	9.50 [7.70–13.85]	13.00 [10.50–17.20]	8.80 [7.80–11.50]	0.01
Angle, ° (median [IQR])	64.00 [52.97–69.22]	66.65 [53.95–70.03]	56.00 [46.40–61.40]	73.70 [67.10–79.10]	< 0.001
Maximum amplitude, mm (median [IQR])	59.50 [49.30–65.50]	60.00 [50.10–65.60]	54.10 [44.40–60.30]	67.65 [65.55–71.08]	< 0.001
CFF					
Maximum amplitude, mm (median [IQR])	21.10 [14.75–29.60]	21.05 [15.12–29.40]	20.00 [14.30–25.90]	41.70 [29.30–55.70]	0.035

*Note:* Heparin dose was calculated as the infusion rate (units/kg/h). A value of 0 units/kg/h indicates patients intentionally managed without systemic heparinization, which is generally associated with cases of bleeding or high hemorrhagic risk.

Abbreviations: APTT, activated partial thromboplastin time; AT3, antithrombin III; CFF, citrated functional fibrinogen; CK, citrated kaolin; CKH, citrated kaolin with heparinase; CRT, citrated rapid thrombelastography; IQR, interquartile range; LY30, lysis at 30 min; PT‐INR, prothrombin time–international normalized ratio; VHA, viscoelastic hemostatic assay.

Table 3 summarizes the ECMO‐related complications stratified by cluster classification. The incidence of thrombotic complications within 24 h before or after VHA measurement did not differ significantly among the clusters. Similarly, no statistically significant differences were found in bleeding events that occurred after VHA.

**TABLE 3:** ECMO‐related complications stratified by the cluster.

	Overall, *n* = 110	Cluster 1, *n* = 76	Cluster 2, *n* = 25	Cluster 3, *n* = 9	*p*
*Thrombosis event*					
Circuit complication before 24 h of VHA measurement, *n* (%)					0.063
Circuit exchange due to thrombosis	2 (1.8)	1 (1.3)	1 (4.0)	0 (0.0)	
Circuit exchange (unknown)	2 (1.8)	1 (1.3)	1 (4.0)	0 (0.0)	
Circuit exchange due to coagulopathy	6 (5.5)	4 (5.3)	2 (8.0)	0 (0.0)	
Circuit exchange due to plasma leak	1 (0.9)	0 (0.0)	1 (4.0)	0 (0.0)	
Circuit exchange due to pump failure	1 (0.9)	0 (0.0)	0 (0.0)	1 (11.1)	
None	98 (89.1)	70 (92.1)	20 (80.0)	8 (88.9)	
Circuit complication after 24 h of VHA measurement, *n* (%)					0.519
Circuit exchange due to thrombosis	1 (0.9)	0 (0.0)	1 (4.0)	0 (0.0)	
Circuit exchange (unknown)	6 (5.5)	5 (6.6)	1 (4.0)	0 (0.0)	
Circuit exchange due to coagulopathy	2 (1.8)	1 (1.3)	1 (4.0)	0 (0.0)	
Circuit exchange due to plasma leak	1 (0.9)	0 (0.0)	1 (4.0)	0 (0.0)	
None	99 (90.0)	69 (90.8)	21 (84.0)	9 (100.0)	
Circuit exchange due to pump failure	1 (0.9)	1 (1.3)	0 (0.0)	0 (0.0)	
*Bleeding event*					
Bleeding event before 24 h of VHA, *n* (%)					0.737
Type 0	82 (74.5)	54 (71.1)	20 (80.0)	8 (88.9)	
Type 1	22 (20.0)	16 (21.1)	5 (20.0)	1 (11.1)	
Type 2	5 (4.5)	5 (6.6)	0 (0.0)	0 (0.0)	
Type 3	1 (0.9)	1 (1.3)	0 (0.0)	0 (0.0)	
Type 4	0 (0.0)	0 (0.0)	0 (0.0)	0 (0.0)	
Site of bleeding before 24 h of VHA, *n* (%)					0.566
Cannulation site	12 (10.9)	10 (13.2)	2 (8.0)	0 (0.0)	
Lower digestive tract	6 (5.5)	6 (7.9)	0 (0.0)	0 (0.0)	
Upper digestive tract	1 (0.9)	0 (0.0)	1 (4.0)	0 (0.0)	
Lung	6 (5.5)	3 (3.9)	2 (8.0)	1 (11.1)	
Surgical site (perianal)	1 (0.9)	1 (1.3)	0 (0.0)	0 (0.0)	
Surgical site (chest wall)	2 (1.8)	2 (2.6)	0 (0.0)	0 (0.0)	
Bleeding event after 24 h of VHA, *n* (%)					0.662
Type 0	83 (75.5)	56 (73.7)	18 (72.0)	9 (100.0)	
Type 1	20 (18.2)	14 (18.4)	6 (24.0)	0 (0.0)	
Type 2	6 (5.5)	5 (6.6)	1 (4.0)	0 (0.0)	
Type 3	1 (0.9)	1 (1.3)	0 (0.0)	0 (0.0)	
Type 4	0 (0.0)	0 (0.0)	0 (0.0)	0 (0.0)	
Site of bleeding after 24 h of VHA, *n* (%)					0.086
Cannulation site	11 (10.0)	9 (11.8)	2 (8.0)	0 (0.0)	
Lower digestive tract	5 (4.5)	5 (6.6)	0 (0.0)	0 (0.0)	
Lung	6 (5.5)	1 (1.3)	5 (20.0)	0 (0.0)	
Other intramuscular hemorrhage	1 (0.9)	1 (1.3)	0 (0.0)	0 (0.0)	
Surgical site (perianal)	2 (1.8)	2 (2.6)	0 (0.0)	0 (0.0)	
Surgical site (chest wall)	2 (1.8)	2 (2.6)	0 (0.0)	0 (0.0)	

Abbreviations: ECMO, extracorporeal membrane oxygenation; VHA, viscoelastic hemostatic assay.

## 4 Discussion

We conducted hierarchical clustering using VHA‐derived and conventional coagulation parameters in patients receiving V‐V ECMO and identified three distinct coagulation phenotypes. Cluster 1 demonstrated a concordant relationship between APTT and CK‐R values, suggesting that APTT control is effective in controlling CK‐R. In contrast, clusters 2 and 3 exhibited prolonged CK‐R relative to APTT. Cluster 2 was characterized by low CK‐MA, suggesting ECMO‐related coagulopathy, whereas cluster 3 showed elevated fibrinogen and platelet counts. Cluster 3 had the highest rate of heparin administration. There were no significant differences in the incidence of thrombotic or bleeding events occurring within 24 h before or after VHA measurements across the three groups and the ICC values suggested that Clusters 1 and 2 reflected dynamic, time‐varying coagulation states, whereas Cluster 3 represented a stable, patient‐specific phenotype. The coexistence of patients that contributed data to multiple clusters while others remained within a single cluster suggests that the clustering method captured both transient and patient‐specific coagulation characteristics. Specifically, Clusters 1 and 2 likely represented time‐varying, state‐dependent coagulation patterns that were influenced by dynamic clinical changes, whereas Cluster 3 reflected a more stable, patient‐specific phenotype. These findings support the concept that ECMO‐associated coagulopathy may involve both dynamic state‐dependent changes and more stable patient‐specific patterns. Cluster 1 likely reflects balanced anticoagulation under standard heparin dosing, while Cluster 2 may represent a state in which cautious heparin reduction or factor supplementation could be considered if validated in future studies, and Cluster 3 is associated with a hypercoagulable tendency that may warrant closer monitoring of the anticoagulation intensity. These interpretations remain speculative but illustrate how VHA phenotyping might inform hypothesis generation for individualized anticoagulation strategies.

Previous studies have reported an association between elevated APTT and bleeding complications in patients receiving ECMO [32, 33]. However, APTT reflects only plasma‐based coagulation factors and may not capture cellular or other elements of coagulation [34]. In contrast, VHA, which utilizes whole blood, provides a more comprehensive assessment of clot formation and stability [19]. The results of VHA, particularly CK‐R, provide evidence for adjusting heparin for coagulation management in patients receiving ECMO [35]. Our findings revealed that patients with comparable APTT values showed substantial variations in CK‐R, highlighting the limitations of APTT in detecting clinically relevant coagulopathies that are better captured by VHA.

Cluster 2 was characterized by prolonged CK‐R and low CK‐MA, which may reflect heparin‐independent coagulopathy, possibly due to consumptive changes or factor deficiencies. Flat‐line patterns are more likely to occur in the setting of profound coagulation factor deficiency, such as in liver failure or massive bleeding [20, 36].

In the comparison among the three groups, platelet counts and fibrinogen levels showed statistical differences. However, the values in clusters 1 and 2 remained within the normal range, whereas those in cluster 3 were markedly elevated. These findings support the notion that, despite measurable differences, conventional markers may not fully capture functional coagulopathy, which can be detected more precisely using VHA.

In our analysis, cluster 3 exhibited substantial elevations in fibrinogen levels and platelet counts, along with prolonged CK‐R relative to APTT. CRT‐MA was highest among the three clusters, further suggesting a hypercoagulable state [37]. These features may reflect inflammation‐associated hypercoagulability, which is potentially associated with COVID‐19 [38]. In cases where hypercoagulability is present without a corresponding prolongation of APTT, anticoagulation is often escalated using higher doses of heparin. Our data consistently showed that cluster 3 had the highest heparin dosage. Although this group may carry a potential risk of bleeding, we observed no significant differences in thrombotic or bleeding complications within 24 h after VHA measurement. Furthermore, the occurrence of flat‐line tracings was limited to one case (11%), suggesting that high‐dose heparin use in this context may be clinically acceptable. While no statistically significant differences in the thrombotic or bleeding outcomes were detected across the clusters, this likely reflects the limited sample size and short observation windows rather than an absence of biological relevance. The identification of distinct coagulation states itself highlights the potential for VHA to capture pathophysiologic heterogeneity that is not discernible from conventional testing, underscoring the need for prospective validation in larger cohorts.

This study has some limitations. First, this was a single‐center retrospective study with a relatively small sample size. In addition, the determination and timing of VHA testing were not standardized and were left to the discretion of the treating clinicians; moreover, not all patients supported with V‐V ECMO underwent the examination. This may have introduced a selection bias toward patients with greater clinical complexity or bleeding concern and may have limited the reproducibility of our findings. Consequently, patients with relatively stable coagulation profiles may have been underrepresented, which could have influenced the observed distribution of coagulation phenotypes.

Second, although latent class mixed models (LCMMs) are commonly used for trajectory‐based phenotyping, our analysis focused on state‐based clustering of VHA measurements rather than patient‐level trajectories. Only a small number of observations were obtained for each patient, and the observations were irregularly spaced; thus, applying LCMMs would likely have resulted in unstable parameter estimation or spurious small latent classes [39, 40]. Therefore, hierarchical clustering was considered a more suitable and interpretable approach for this dataset. However, multiple VHA measurements were obtained from the same patients, suggesting that the assumption of statistical independence underlying the ANOVA and Kruskal–Wallis tests may not be fully met. This limitation should be considered when interpreting the comparative analyses, and future studies employing mixed‐effects or longitudinal modeling are warranted to account for within‐patient correlations. Third, although the anti‐Xa assay is considered the gold standard for monitoring UFH, it was not available in this study because of limitations in national insurance coverage and accessibility in Japan. This limitation may have also influenced the distribution of coagulation phenotypes, as heparin activity could not be fully characterized without anti‐Xa monitoring. Fourth, thrombotic and bleeding complications were assessed only within a short timeframe, potentially underestimating clinically important events occurring outside this window, including delayed bleeding or thrombotic events. Longitudinal data describing the bleeding and thrombotic events across the entire ECMO course were not systematically collected in this retrospective dataset, and future studies should incorporate comprehensive outcome tracking to address this limitation. Fifth, this study exclusively focused on patients receiving V‐V ECMO. Given the differences in coagulation profiles and anticoagulation strategies between the ECMO modalities, our findings may not be generalizable to patients supported by venoarterial ECMO or other mechanical circulatory support systems. Finally, because this was an observational study, causal relationships could not be established. Nevertheless, our findings highlight the potential value of VHA as a complementary tool for conventional coagulation tests and a possible role in informing individualized anticoagulation management during V‐V ECMO. In particular, individual VHA measurements may help characterize the dynamic coagulation states and support more informed transfusion decision‐making. Taken together, these phenotypes may represent theoretically distinct anticoagulation contexts during ECMO, including relatively balanced anticoagulation, consumptive or factor‐related coagulopathy, and a hypercoagulable inflammatory state.

In conclusion, hierarchical clustering analysis revealed heterogeneity in coagulation phenotypes among patients receiving V‐V ECMO that could not be clearly distinguished using conventional laboratory tests alone. Three distinct phenotypes were identified. Cluster 1 demonstrated concordant APTT and CK‐R prolongation, Cluster 2 exhibited disproportionate CK‐R prolongation with low clot strength, and Cluster showed a hypercoagulable profile with elevated fibrinogen and platelet counts despite prolonged CK‐R. These findings suggest that VHAs may complement APTT monitoring by identifying coagulation abnormalities not captured by conventional testing during VV‐ECMO management. The identification of distinct coagulation phenotypes highlights the potential for VHA‐guided risk stratification and individualized anticoagulation strategies in this population. However, these phenotypes should be considered exploratory, and their clinical utility requires validation through prospective studies with protocolized VHA assessment and systematic outcome tracking. Future research should focus on defining optimal VHA thresholds, establishing treatment algorithms for each phenotype, assessing longitudinal trends in coagulation status using mixed‐effects approaches, and evaluating whether phenotype‐directed anticoagulation management improves clinical outcomes compared with standard APTT‐based approaches.

## Author Contributions

Daisuke Irimada: conceptualization, methodology, formal analysis, and writing. Yudai Iwasaki: conceptualization, data curation, formal analysis, writing – original draft. Yusuke Takei: methodology, writing – review and editing. Moe Oyama, Ami Tanaka, Shinichi Yamaguchi, Naoya Kobayashi, Daisuke Konno, and Takuya Shiga: conceptualization, writing – review and editing. Masanori Yamauchi: writing – review and editing, supervision, project administration.

## Funding

This work, including English language editing, was supported by the JSPS KAKENHI (Grant JP23K15609 [to Yusuke Takei]).

## Conflicts of Interest

The authors declare no conflicts of interest.

## Supporting information


**Figure S1:** aor70153‐sup‐0001‐SupplementalMaterials.docx.

## Data Availability

The data that support the findings of this study are available from the corresponding author upon reasonable request.
