# Supplementary material for: Identification of Coagulation Phenotypes Using Viscoelastic and Conventional Assays During Veno‐Venous Extracorporeal Membrane Oxygenation: A Hierarchical Clustering Analysis
Source: Artif Organs. 2026 Apr 21;50(8):1170–81. doi: 10.1111/aor.70153 (PMC13452916; doi:10.1111/aor.70153)
Supplement: Supplementary file 1 [file AOR-50-1170-s001.docx]

Supplemental Figure 1: Time plot of VHA measurement for each patient

Supplemental Figure 2:

(A)	Elbow method for optimal K

(B)	Gap statistical analysis

Supplemental Figure 3:
(A) Hierarchical clustering dendrogram constructed using Ward’s method, in which the structure of the clusters is illustrated based on coagulation parameters.

(B) Cluster classification of coagulation profiles visualized in two-dimensional space using principal component analysis (PCA). Three distinct clusters were identified.

Supplemental Figure.4: Time plot of VHA measurement for each patient divided by cluster
